# An analysis of the relationship between Glasgow Coma Scale score and plasma glucose level according to the severity of hypoglycemia

**DOI:** 10.1186/2052-0492-2-1

**Published:** 2014-01-03

**Authors:** Atsushi Kotera, Shinsuke Iwashita, Hiroki Irie, Junichi Taniguchi, Shunji Kasaoka, Yoshihiro Kinoshita

**Affiliations:** Department of Emergency and General Medicine, Kumamoto University Hospital, 1-1-1 Honjo, Chuo-ku, Kumamoto City, Kumamoto Prefecture, 860-8556 Japan; Department of Intensive Care Unit, Kumamoto University Hospital, 1-1-1 Honjo, Chuo-ku, Kumamoto City, Kumamoto Prefecture, 860-8556 Japan

**Keywords:** Hypoglycemia, Glasgow Coma Scale, Diabetes mellitus

## Abstract

**Background:**

The Glasgow Coma Scale (GCS) score of an individual with hypoglycemia is expected to be low due to an insufficient glucose supply to the brain. However, we sometimes encounter hypoglycemic patients with high GCS scores. This study was undertaken to analyze the relationship between the GCS score and the plasma glucose level.

**Methods:**

Among the patients with neurological impairments admitted to our institution between October 1, 2010 and March 31, 2013, the cases of 41 hypoglycemic patients were examined in this retrospective cohort study. The defined plasma glucose level for mild hypoglycemia was 41–60 mg/dL, that for moderate hypoglycemia was 21–40 mg/dL, and that for extreme hypoglycemia was below 20 mg/dL. We divided the patients into two groups: those with mild hypoglycemia (*n* = 14) and those with moderate/extreme hypoglycemia (*n* = 27). We compared the two groups' physiological data and assessed the relationship between the GCS score and the plasma glucose level by Spearman rank correlation (*ρ*), the significance of which was determined by Spearman's rank sum test. We used the Mann-Whitney *U*-test and the chi-square (*χ*^2^) test to test for differences between the two groups when appropriate.

**Results:**

Three hundred twenty-six patients with neurological impairments were admitted during the study period, and 41were eligible hypoglycemic patients. The GCS scores of the 14 patients with mild hypoglycemia were significantly higher than those of the 27 patients with moderate or extreme hypoglycemia (median score 12, range 7–15 vs. 10, 3–15, *p* = 0.0367). There were no significant differences in physiological data (including autonomic symptoms) between the two groups. Spearman's rank sum test was 0.491 in the total group of 41 hypoglycemic patients, 0.053 in the mild hypoglycemic patients, and 0.493 in the moderately or extremely hypoglycemic patients.

**Conclusions:**

The relationship between the GCS score and the plasma glucose level differed according to the severity of hypoglycemia. Even when a patient has a high GCS score, careful assessment of differential diagnosis should be conducted and the possibility of hypoglycemia should be considered in light of his or her neurogenous symptoms.

## Background

The Glasgow Coma Scale (GCS) was developed to describe the level of consciousness in patients with traumatic brain injury [1]. It measures the patients' best eye, motor, and verbal responses, and it classifies the level of consciousness as mild (score of 14–15), moderate (9–13), or severe (3–8) [2]. The GCS is also used for describing the level of consciousness in hypoglycemic patients [3], and it is widely accepted as a tool for both trauma and non‒trauma patients.

Hypoglycemic patients commonly have neuroglycopenic symptoms. The GCS score of hypoglycemic patients is predicted to be low because the glucose supply to the brain is insufficient. However, we sometimes encounter the hypoglycemic patients with high GCS scores. There are few reports concerning the relationship between the GCS score and the plasma glucose level in hypoglycemic patients. In the present study, we analyzed the relationship between these two parameters according to the severity of hypoglycemia and compared the physiological data among patients with differing degrees of hypoglycemia.

## Methods

### Patients

We investigated patients age 18 and over who were transferred to our institution with neurological impairments between October 1, 2010 and March 31, 2013. Among those patients, only the hypoglycemic patients were eligible for the present study. Hypoglycemia was defined as a plasma glucose level below 60 mg/dL, accompanied by a wide variety of symptoms [4]. We also used the following definitions: mild hypoglycemia, a plasma glucose level of 41–60 mg/dL; moderate hypoglycemia, 21–40 mg/dL; extreme hypoglycemia, below 20 mg/dL [4]. We usually perform the sampling of either arterial blood or capillary blood rapidly and check the glucose level by arterial blood gas analyzer or glucose meter. The choice of the material and the device is committed to each emergency physician. On the other hand, we also perform the sampling of venous blood without delay, and the plasma glucose level per patient is measured by glucose oxidase method in a central laboratory. Thus, in the present study, we used the sample of venous blood.

### Protocol design

This was a retrospective cohort study. All data were obtained from medical charts and did not include any personal information that would identify any of the patients. Therefore, informed consent from the patients was waived, based on the Ethical Guidelines for Epidemiological Studies issued jointly by the Ministry of Health, Labour and Welfare and the Ministry of Education, Culture, Sports, Science, and Technology of Japan.

### Data collection

The data collected from medical charts were age, gender, symptoms, GCS score, renal dysfunction, diabetes mellitus (DM) status, body temperature, heart rate, systolic blood pressure, and plasma glucose level. Lucidity was defined as GCS score of 15, mild confusion was GCS of 14, moderate confusion was GCS of 9–13, and severe confusion or coma was GCS of 3–8 [2]. Estimation of the level of consciousness by the GCS score and sampling of the venous blood to measure the plasma glucose level were performed at the same time as part of the Emergency Room visit.

We divided the patients into two groups: the mild hypoglycemia group and the moderate/extreme hypoglycemia group. The physiological data of the two groups were compared. Renal dysfunction was defined as a glomerular filtration rate (GFR) less than 60 mL/min [5]. In our study, the GFR was assessed by determining the estimated GFR. DM was defined according to the World Health Organization criteria as the presence of either fasting venous plasma glucose levels of 7.0 mmol/L (126 mg/dL) or greater, or 2-h venous plasma glucose levels of 11.1 mmol/L (200 mg/dL) or greater, or a 75 g oral glucose tolerance test result [6].

### Statistical analysis

All statistical analyses were performed using the software programs SPSS 17.0 (SPSS, Chicago, IL, USA) and Excel Tokei 2012 (Social Survey Research Information Co., Tokyo, Japan). Intergroup differences were assessed with the *χ*^2^ test with Yates' correlation for continuity in categorical variables. The Mann-Whitney *U* test was used to test for differences in continuous variables. We considered *p* values <0.05 significant. We analyzed the correlation between the GCS score and plasma glucose level by Spearman rank correlation (*ρ*), the significance of which was determined by Spearman's rank sum test.

## Results

The number of patients with neurological impairments admitted to our institution during the study term was 326 of the 3,514 patients, and 41 of these 326 patients were hypoglycemic. The median plasma glucose level in the 41 patients was 32 mg/dL (range, 10–58 mg/dL). The number of patients with mild hypoglycemia was 14, that of patients with moderate hypoglycemia was 21, and that of patients with extreme hypoglycemia was 6. Because the number of patients with extreme hypoglycemia was small, we divided the patients into the two groups of mild hypoglycemia and moderate/extreme hypoglycemia.

The symptoms of the two groups were shown in Table 1. The symptoms in the mild hypoglycemia group were confusion in ten patients (two mild, seven moderate, and one severe), difficulty in speech in one patient, weakness in two patients, and convulsions in one patient. The symptoms in the moderate/extreme hypoglycemia group were confusion in 26 patients (4 mild, 12 moderate, and 10 severe) and weakness in 1 patient. Several patients complained of various neurological impairments besides confusion.

**Table 1:** **The symptoms on arrival between the two groups**

	Mild hypoglycemia group	Moderate/extreme hypoglycemia group	Total
Confusion	10	26	36
Mild (GCS score of 14)	2	4^b^	6
Moderate (GCS score of 9–13)	7^a^	12^c^	19
Severe (GCS score of 3–8)	1	10^d^	11
Difficulty in speech	1	-	1
Weakness	2	1	3
Convulsions	1	-	1
Total	14	27	41

^a^In the seven patients, difficulty in speech, convulsions, or weakness was observed in each patient; ^b^in the four patients, difficulty in speech or left hemiplegia was observed in each patient; ^c^in the 12 patients, 1 had convulsions; ^d^in the ten patients, two had convulsions.

The causes of hypoglycemia in the two groups were shown in Table 2. Of the 41 patients, 33 patients had DM, 24 were treated with insulin, and 9 were treated with oral anti‒diabetic agents. Ten patients had insulin‒dependent DM, and 23 patients had non-insulin‒dependent DM. The common causes of hypoglycemia in the diabetic patients were low calorie intake due to illness or dieting (23/33) and suspected overdose of insulin or oral anti‒diabetic agents (5/33). The causes in the non‒diabetic patients were various: malnutrition due to chemotherapy in two patients, and anorexia nervosa, hepatocellular carcinoma secreting insulin-like growth factor II, sudden withdrawal from long-term steroid treatment, dumping syndrome, and alcoholism (one patient each). In the mild hypoglycemia group, 11 patients had DM and the other 3 did not. The moderate/extreme hypoglycemia group included 22 patients with DM and 5 without.

**Table 2:** **The causes of hypoglycemia in the two groups**

	The causes of hypoglycemia	Mild hypoglycemia group	Moderate/extreme hypoglycemia group	Total
With DM (*N* = 33)	Low calorie intake due to illness or dieting	7	16	23
	Over dose of anti-diabetic therapy	2	3	5
	Malnutrition due to chemotherapy	1	2	3
	Unclear	1	1	2
Without DM (*N* = 8)	Malnutrition due to chemotherapy	-	2	2
	Anorexia nervosa	-	1	1
	Hepatocellular carcinoma secreting ILGF II	-	1	1
	Withdrawal from long-term steroid therapy	1	-	1
	Dumping syndrome	1	-	1
	Alcoholism	-	1	1
	Unclear	1	-	1
Total		14	27	41

*DM* diabetes mellitus, *ILGF II* insulin-like growth factor II.

The groups' physiological data (in medians and ranges) are provided in Table 3. There were no significant differences between the two hypoglycemia groups in age, gender, renal dysfunction, rate of DM, body temperature, heart rate, or systolic blood pressure. However, the GCS scores in the mild hypoglycemia group were significantly higher than those in the moderate/extreme hypoglycemia group (median GCS score 12, range 7–15, vs. 10, 3–15, *p* = 0.0367).

**Table 3:** **The physiological data on admission between the two groups**

	Mild hypoglycemia group	Moderate/extreme hypoglycemia group	***p*** value
Plasma glucose level (mg/dL)	47 (42–58)	28 (10–40)	-
Age (years)	65 (29–95)	67 (26–84)	0.4828
Gender	Male/female = 8:6	Male/female = 18:9	0.5483
Estimated GFR (mL/min)	53 (8–90)	67 (10–90)	0.2405
Diabetes mellitus status	11/14 (91%)	22/27 (76.7%)	0.8236
GCS score	12 (7–15)	10 (3–15)	0.0367
Body temperature (°C)	36.1 (34.8–36.6)	35.9 (29.6–37.3)	0.2648
Heart rate (beats/min)	87 (48–127)	86 (62–126)	0.8044
Systolic blood pressure (mmHg)	137 (90–198)	138 (92–198)	0.9561

*GFR* glomerular filtration rate, *GCS* Glasgow Coma Scale.

**Figure 1:**
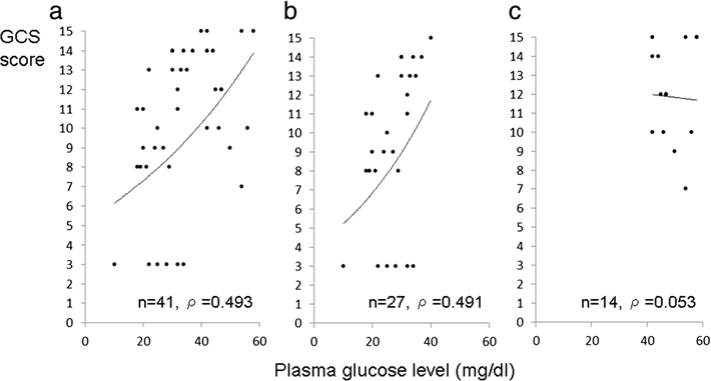
**Scatter graph demonstrating the relationship between the GCS scores and the plasma glucose levels. (a)** The value of the Spearman's rank sum test was statistically high (*ρ* = 0.493) in the total hypoglycemic patients. **(b)** The value of the Spearman's rank sum test was statistically high (*ρ* = 0.491) in the moderate or extreme hypoglycemic patients. **(c)** The value of the Spearman's rank sum test was low (*ρ* = 0.053) in the mild hypoglycemic patients.

**Figure 2:**
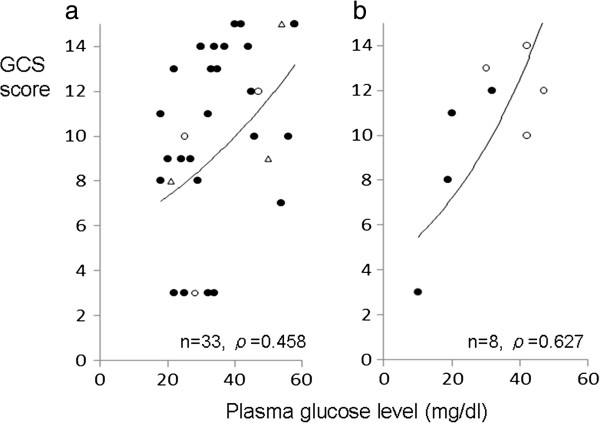
**Scatter graph demonstrating the relationship between the GCS scores and the plasma glucose levels.** Closed circles indicate the patients who had recurrent hypoglycemic episodes. Open circles indicate the patients who had no hypoglycemic episodes. Open triangles indicate the patients whose hypoglycemic episodes were unclear. **(a)** The value of the Spearman's rank sum test was statistically high (*ρ* = 0.458) in the diabetic patients. **(b)** The value of the Spearman's rank sum test was high (*ρ* = 0.627) in the non-diabetic patients, but not significant.

Figures 1 and 2 demonstrate the relationship between the GCS scores and the plasma glucose levels. The GCS scores were significantly correlated with the plasma glucose levels in the total hypoglycemic patients (Figure 1a, *ρ* = 0.491, *p* < 0.01) and in the moderate/extreme hypoglycemia group (Figure 1b, *ρ* = 0.493, *p* < 0.01). However, in the mild hypoglycemia group, the GCS scores were not correlated with plasma glucose levels (Figure 1c, *ρ* = 0.053, *p* = 0.857). The relationship was significant in the diabetic patients (Figure 2a, *ρ* = 0.458, *p* < 0.05), but not significant in the non‒diabetic patients; nevertheless, the value of Spearman's rank sum test was high (Figure 2b, *ρ* = 0.627, *p* = 0.096).

## Discussion

The relationship between the GCS scores and the plasma glucose levels was significant in the total hypoglycemic patients and in the moderate/extreme hypoglycemia group. However, the number of patients in the mild hypoglycemia group is small and the relationship was not significant. We considered the mechanisms that might underlie this discrepancy.

In general, neuroglycopenic symptoms are observed when an individual's plasma glucose level reaches approximately 50–60 mg/dL [7]. It was reported that the plasma glucose level at which cognitive dysfunction was observed was approximately 57 mg/dL [8], the level for neurogenous symptoms was approximately 55 mg/dL, the level for disorders of mental function was 46 mg/dL, and the level for convulsions or coma was 27 mg/dL [9]; however, these levels varied among the patients [9]. Of the strictly regulated diabetic patients in that study, almost all had experienced hypoglycemia previously, and the plasma glucose levels at the onset of neuroglycopenic symptoms might have declined further [9]. In addition, recurrent hypoglycemic episodes can weaken the adrenalin and glucagon response to decreased plasma glucose levels, and severe hypoglycemia can be induced [9]. This weakened response was recently named hypoglycemia-associated autonomic failure (HAAF), and it was described as a potential risk factor for ‘hypoglycemia unawareness’ [9–11]. Conversely, in the poorly controlled diabetic patients of the same study, the plasma glucose levels at the onset of neuroglycopenic symptoms became higher [9, 12].

In the 33 diabetic patients, 27 had recurrent hypoglycemic episodes, 3 had no episodes, and 3 were unclear. The median (range) GCS scores and plasma glucose levels in the diabetic patients with versus (vs.) without recurrent hypoglycemic episodes were 11 (3–15) and 33 mg/dL (18–58 mg/dL) vs. 10 (3–12) and 28 mg/dL (25–47 mg/dL) (not significant). The number of diabetic patients without recurrent hypoglycemic episodes was small, and it was difficult to elucidate the effect of recurrent hypoglycemic episodes statistically in the diabetic patients.

The hemoglobinA1c (HbA1c) level indicates whether DM is well controlled or not, and it reflect the patients' glycemic condition for the prior 3–4 months [13]. Unfortunately, the HbA1c within 3–4 months of the date on which a hypoglycemic episode occurred was not measured in 9 of the 33 diabetic patients. The median (range) HbA1c level was 6.3% (5.3%–11.8%); however, this level did not reflect the data of all the diabetic patients, and the analysis was not enough to predict whether DM was well controlled or not.

On the other hand, four of the nine patients treated with oral anti‒diabetic agents were medicated with two or three kinds of anti‒diabetic agents. The median (range) daily insulin dosage was 0.56 U/kg (0.28–1.14 U/kg) in the 24 patients treated with insulin, and the dosage was equivalent to that in other studies [14, 15]. Indeed, hypoglycemia induced by the overdose of insulin therapy or oral anti‒diabetic agents was observed in five patients, and almost all of the diabetic patients had experienced recurrent hypoglycemic episodes. Furthermore, the median plasma glucose level in the diabetic patients of the present study was lower than that in the poorly controlled diabetic patients of the other study (33 vs. 78 mg/dL) [12]. Considering these data, it appears that almost all of the diabetic patients in the present study are thought to be strictly controlled.

In the eight non‒diabetic patients, four had recurrent hypoglycemic episodes and four did not have. The median (range) GCS scores and plasma glucose levels in the patients with vs. without recurrent hypoglycemic episodes were 10 (3–12) and 20 mg/dL (10–32 mg/dL) vs. 13 (10–14) and 42 mg/dL (30–47 mg/dL). The plasma glucose levels were significantly lower in the patients with recurrent hypoglycemic episodes (*p* = 0.0421), but there was no significant difference in the GCS scores. The number of patients is small; however, we can construct the hypothesis that the plasma glucose levels at the onset of neuroglycopenic symptoms decline due to recurrent hypoglycemic episodes in the non‒diabetic patients.

The recurrent hypoglycemic episodes and the strict management for DM would shift the plasma glucose levels at the onset of neuroglycopenic symptoms to the lower levels, and that may have contributed to the discrepancy in the Spearman's rank sum test. When the plasma glucose levels were 41–60 mg/dL, the levels were not low enough to cause severe neurogenous symptoms and the GCS scores did not correlate with the plasma glucose levels. Conversely, when the plasma glucose levels were below 40 mg/dL, the levels were low enough to cause severe neurogenous symptoms in almost all of the patients and the GCS scores were significantly correlated with the plasma glucose levels. The cause of the declined plasma glucose levels at the onset of neuroglycopenic symptoms might be different between the diabetic and non‒diabetic patients; however, the high frequency of moderate or extreme hypoglycemia had contributed to the high value of Spearman's rank sum test in the diabetic and non-diabetic patients. In the future, even when a patient has a high GCS score, a careful assessment of differential diagnosis should be made, and the treating physicians should consider the possibility of hypoglycemia in light of the patient's various neurogenous symptoms.

Hypoglycemia also causes autonomic symptoms that show dependence on the according to plasma glucose levels [7, 9]. The increased levels of glucagon and adrenaline could cause tachycardia or hypertension. The reported plasma glucose levels of each autonomic symptom were as follows: increased glucagon secretion at 69 mg/dL, increased adrenalin secretion at 66 mg/dL, and increased cortisol secretion at 57 mg/dL [9]. There were no significant differences in heart rate or systolic blood pressure between the two groups. Heart rate or systolic blood pressure was influenced by anti‒hypertensive drugs. Indeed, in the present study, 24 of the 41 patients were medicated with anti‒hypertensive drugs. The level of adrenalin, glucagon, and cortisol were not measured in our patients, and we could not detect any differences in autonomic symptoms according to the plasma glucose levels. We will conduct such an analysis in the future.

There are several limitations in this study. The sample size of patients was small, and the data reflect the experience of a single center. Thus, the results may not be generalized for the population as a whole. Ongoing trials and reexamination with a large number of patients at several centers are necessary to test the results in the present study. In addition, the study was a retrospective investigation. Further examinations are needed to evaluate the results after measuring the adrenaline, glucagon, and cortisol levels in hypoglycemic patients. Finally, Inoue et al. [16] reported the difference of the accuracy of blood-glucose measurements between arterial blood gas analyzers and glucose meters by using arterial blood or capillary blood. It should be noted that a choice of the blood sample or the device could influence the blood glucose levels.

## Conclusions

The relationship between the GCS scores and the plasma glucose levels differed according to the severity of the patients’ hypoglycemia. Even when a patient has a high GCS score, his or her differential diagnosis should be carefully evaluated and the possibility of hypoglycemia should be considered in light of various neurogenous symptoms.
